# On the Relationship Between Well-Being and Exercise Adherence for Children and Adolescents: A Systematic Mini Review

**DOI:** 10.3389/fpsyg.2022.900287

**Published:** 2022-05-23

**Authors:** Jiping Chen, Chenggang Wu

**Affiliations:** ^1^Faculty of Physical Education, Shandong University, Jinan, China; ^2^Key Laboratory of Multilingual Education With AI, School of Education, Shanghai International Studies University, Shanghai, China

**Keywords:** well-being, exercise adherence, children, adolescents, positive effects

## Abstract

Although the close positive relationship between well-being and exercise adherence has been confirmed by numerous studies, it is still unclear whether this relationship exists for children and adolescents, because previous research mainly focuses on adults. The present review systematically explored the relationship between well-being ranging from individual to social aspects and exercise adherence based on extant studies. Seven studies including both quantitative and qualitative studies were analyzed. The results showed that well-being was not related to exercise adherence as strongly as expected. In some cases, well-being was even negatively associated with exercise adherence. Limited sample size, insensitive measurement of exercise adherence, gender, and mental and physical condition of children and adolescents might partially influence the relationship between well-being and exercise studies. However, the studies at hand are still in their infancy. More studies on the relationship between well-being and exercise adherence are needed for children and adolescents, especially in non-western countries.

## Introduction

Over the years, studies have found a steady decline in the physical health of children and adolescents (Tomkinson et al., 2003; Tomkinson and Olds, 2007; Tremblay et al., 2010). For example, over the past few decades, the prevalence of obesity has increased from 0.7% in 1975 to 5.6% in 2016 for girls and from 0.9% in 1975 to 7.8% in 2016 for boys (NCD Risk Factor Collaboration [NCD-RisC], 2017). There is also a gradual increase in the prevalence of chronic conditions such as type 2 diabetes and depression, which can also pose a big challenge to the physical health of children and adolescents in adulthood (Harhay et al., 2012; Hales et al., 2017). There are some approaches to increase physical health for children and adolescents. Exercise adherence, or exercise persistence, is one of the efficient approaches that can significantly accentuate physical health (Janssen and Leblanc, 2010; Glowacki et al., 2017). Previous studies found that children and adolescents benefited from high exercise adherence by improving their physical and mental health, sleep quality, brain development, skeletal health, and social, psychological, and cognitive health (Brown and Lawton, 1986; Scully et al., 1998; Kirkcaldy et al., 2002; Andersen, 2006). There is ongoing research to support the importance of at least 60 min/day of moderate to vigorous physical activity for disease prevention and health promotion in children and adolescents (Ekelund et al., 2012; Poitras et al., 2016). Therefore, increasing the adherence of children and adolescents to exercise is powerful in preventing and improving physical health. However, poor exercise adherence among youth has been a prominent public health concern (Bruner and Spink, 2011). The problem is evolving in a polarizing trend. For example, recent researchers from the World Health Organization’s Global Trends in Physical Inactivity noted that 80% of the world’s 1.6 million adolescents, do not meet the recommended 1 h of moderate to vigorous activity per day (Guthold et al., 2020), suggesting that only about 20% of adolescents are adhering to 1 h of exercise per day.

Children and adolescents’ adherence to exercise, although not consistently defined, measured or reported, is currently judged in such studies primarily through rates of attendance at exercise training interventions for children and adolescents, where rates range from 56 to >99% (Wong et al., 2008; Davis et al., 2009; Hasson et al., 2012; Lee et al., 2012). Researchers have been conducting a series of studies to facilitate exercise adherence for children and adolescents and noticed positive effects and well-being factors can possibly serve as the facilitators for exercise adherence (Hu et al., 2021). Well-being is a multifaceted concept that ranges from subjective feeling of positive, happy, comfort, and prosperous to experiences and living conditions of being positive and desirable (Ben-Arieh et al., 2014). In the present study, we adopted the concept of well-being at both individual and social aspects. Individual aspect of well-being was mostly related to positive subjective feeling, such as happiness, enjoyment, the meaning of life, satisfaction, hope, whereas social well-being aspects, included social support and coherence, such as encouragement, social support, and accompany. There is evidence showing that well-being can possibly increase exercise adherence for children and adolescents. For example, previous studies found that individual self-esteem, internal pleasure, and social confidence, external encouragement and support in a group exercise perceived health, individual well-being, pleasure, and joy when exercising all significantly promoted exercise adherence of children and adolescents (Bergman et al., 2008; Kallings et al., 2009; Rodrigues et al., 2021). Additional evidence showed that exercise maintenance in the general population depended primarily on motivational, psychological, supportive, and environmental factors (Bélanger et al., 2011; Martins et al., 2015). Although there are some studies on children and adolescents, as introduced above, most of the existing research is on exercise adherence in adults or older people. The current study aimed to systematically explore the extant studies on how exercise adherence is shaped by well-being for children and adolescents.

Given the importance of exercise adherence for developing children and adolescents and the enduring beneficial influences of exercise adherence on later life, the present study aimed to answer the following three research questions.

RQ1: What are the extant studies on the influence of well-being on exercise adherence among children and adolescents?

RQ2: Based on the extant studies, whether there is any association between well-being factors and exercise adherence for children and adolescents.

RQ3: If there is any significant relationship, whether the relationship between well-being factors and exercise adherence is positive or negative.

Exploring RQ1 would identify the well-being factors that have been investigated in exercise adherence for children and adolescents. After identifying the well-being factors that are confirmed to be related to exercise adherence (RQ2 and RQ3), we would delineate possible directions for interventions to enhance exercise adherence for children and adolescents. However, the present study did not explore the amateur or professional athletes and physical education class, because sports and physical activities for athletes and physical education are different from regular physical activities.

In sum, the present mini-review aimed to explore what are the well-being factors that might contribute to exercise adherence and how these factor shape the exercise adherence for children and adolescents.

## Materials and Methods

Several stages of this review followed the recommendations set out in the PRISMA protocol (Moher et al., 2009).

### Searching Strategy

Literature searching was conducted on 19 November 2021 from the following electronic databases: Web of Science, PubMed, SPORTDiscus, and PsycINFO. We did not restrict the time range, because it was attempted to cover as many as extant studies about the positive effect and well-being factors on exercise adherence among children and adolescents. A Boolean search strategy was used to identify articles that included following the keywords: (“exercise adherence” OR “persistence in exercise” OR “exercise persistence”) AND (“children” or “adolescents” or “youth” or “child” or “teenager” or “kids” or “childhood” or “juvenile”) AND (“enjoyment” or “joy” or “fun” or “pleasure” or “positive affect” or “well-being” or “well being” or “happiness” or “satisfaction”).

### Inclusion/Exclusion Criteria

Studies were included based on the following criteria: (1) The study concentrated on children and adolescents; (2) Positive psychological factors were independent variables; (3) The dependent variable should be exercise adherence; (4) The study should be written in English; (5) Full-text was available; (6) The studies were published in scholarly (peer-reviewed) journals; (7) The studies should be empirical.

The studies were excluded if they met the following criteria: (1) Studies focused on amateur or professional athletes, as sport and physical activity are different concepts (Caspersen et al., 1985); (2) Research on physical education classes, as this type of physical activity is different from regular sports (Caspersen et al., 1985); (3) Instrument validation studies; (4) Gray literature; (5) Review studies.

### Study Selection

**FIGURE 1:**
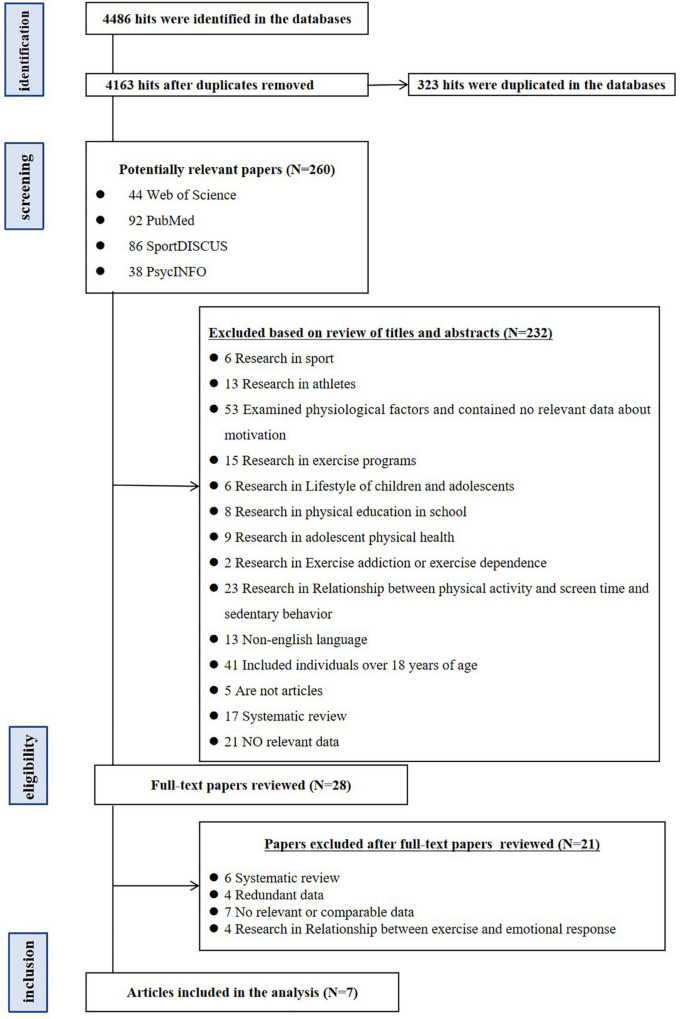
PRISMA flowchart.

After initial searching for the literature (Figure 1), a total of 4,486 titles were identified where 323 articles were found to be duplicates. After filtering the titles of 4,163 articles, 260 studies were selected as being potentially relevant to the present study. After carefully reading the abstracts of the articles, 28 articles were kept for full-text reading. After reading the full text, studies that did not meet the inclusion criteria or met the exclusive criterion were trimmed. Finally, seven articles were kept for the present study.

### Quality of the Studies

Quality assessment for included studies was provided in Supplementary Material. The two authors assessed the included quantitative studies using the questionnaire used in previous studies (Zhang et al., 2022). The qualitative studies were assessed with Joanna Briggs Institute approach (Santos et al., 2018). The studies were of sufficient quality.

## Results

Seven articles (Douthitt, 1994; Bruner and Spink, 2011; Birt et al., 2014; Alberga et al., 2019; Guerrero et al., 2020; Faulkner and Michaliszyn, 2021; Sunesson et al., 2021) were kept for review and related information is shown in Table 1. The results summarized below were presented according to the purposes of the current review.

**TABLE 1:** Description of reviewed studies.

Author (date)	Location	Design	Size (%F)	Features	Measures	Outcomes	Analysis	Main findings
Alberga et al., 2019	Canada	Experimental (22 weeks)	228 (n/a)	Post-pubertal adolescents (15.6 ± 1.4)	Brunel Mood Scale, Harter global self-esteem, children’s self perception of adequacy and predilection for physical activity (CSAPPA), exercise self-confidence survey	Low exercise adherence	Logistic regression	Mood: Vigor (n.s.), self-esteem (n.s.), motivation: predilection (n.s.), enjoyment (n.s.), exercise self-efficacy (n.s.)
Guerrero et al., 2020	Canada	Cross-sectional	1472 (54)	Parents (Mean age = 45.12, SD = 7.55), children aged 5–17 years [aged 5–11 years (47.1%) and 12–17 years (52.9%)]	Self-made questionnaire	Adherence to 24-h movement guidelines	Decision tree models	Parental perceived capability to support children’s physical activity (+), parent support change (n.s.)
Bruner and Spink, 2011	Canada	Experimental (24 days)	122 (n/a)	Youth (aged 13–17 years)	Group task satisfaction (multidimensional athlete satisfaction questionnaire)	Dropout	ANCOVA	Group task satisfaction (n.s.)
Bruner and Spink, 2011	Canada	Experimental (24 days)	122 (n/a)	Youth (aged 13–17 years)	Group task satisfaction (multidimensional athlete satisfaction questionnaire)	Attendance	ANCOVA	Group task satisfaction (n.s.)
Faulkner and Michaliszyn, 2021	United States	Experimental (22 weeks)	21 (71)	Type 2 DM (15.4 ± 2.2), obese (14.8 ± 1.8)	Diabetes social support questionnaire-family version	Daily average MVPA intensity	Correlation/Pearson correlation coefficient	Family support for exercise (n.s.)
Douthitt, 1994	United States	Cross-sectional	132 (28.8)	Students (aged 13–17 years)	The adolescent self-perception profile, the perceived control at school scale, the self-motivation inventory, the psychosocial activity dimensions profile	Exercise adherence (habitual physical activity questionnaire)	Multiple regression	Perceived athletic competency (−) for female, (n.s.) for male
Douthitt, 1994	United States	Cross-sectional	110 (30)	Students (aged 13–17 years)	The adolescent self-perception profile, the perceived control at school scale, the self-motivation inventory, the psychosocial activity dimensions profile	Exercise adherence (habitual physical activity questionnaire)	Multiple regression	Perceived global self-worth (−) for female, (n.s.) for male
Sunesson et al., 2021	Sweden	Qualitative study	14 (71)	Major depressive disorder (13–17 years)	Semi-structured interviews	Low exercise adherence	Content analysis	Self-esteem (+), a supportive environment (+)
Birt et al., 2014	United Kingdom	Qualitative study	19 (53)	Children and young people (9–17 years)	Semi-structured interviews	Adherence to 24-h movement guidelines	Thematic analysis	Parental supervision (+), family support (−)

*+, means positive correlation; −, means negative correlation; n.s., means non-significant correlation.*

The first aim of this mini-systematic review was to find existing studies on the influence of well-being on exercise adherence in children and adolescents, as these existing studies could identify individual and social well-being factors that can influence exercise adherence in children and adolescents. As for approaches, the seven articles included both quantitative (*N* = 5) and qualitative studies (*N* = 2). Of the quantitative studies, there were four experimental studies and one cross-sectional correlational studies. The studies all used self-report questionnaires and surveyed a total of 2,085 children and adolescents. The participants in the four studies were healthy children and adolescents (Douthitt, 1994; Bruner and Spink, 2011; Alberga et al., 2019; Guerrero et al., 2020). Further, Faulkner’s study explored the children and adolescents with obesity and type 2 diabetes (Faulkner and Michaliszyn, 2021). The qualitative studies all used semi structured interviews, with 19 healthy children and adolescents in Birt’s study and 14 children and adolescents with depression in Sunesson’s study (Birt et al., 2014; Sunesson et al., 2021). Of the seven studies included, only one examined both individual and social positive psychological factors influencing children and adolescents’ exercise adherence (Sunesson et al., 2021). The remaining studies focused on a single dimension of positive psychological factors influencing exercise adherence in children and adolescents (Douthitt, 1994; Bruner and Spink, 2011; Birt et al., 2014; Alberga et al., 2019; Guerrero et al., 2020; Faulkner and Michaliszyn, 2021).

Another aim of this mini-systematic review was to examine how exercise adherence in children and adolescents was influenced by well-being factors (RQ2 and RQ3).

For quantitative studies, eight individual well-being factors were investigated in the study of the relationship between well-being and exercise adherence for children and adolescents, including vigor, self-esteem, predilection, enjoyment, exercise self-efficacy (Alberga et al., 2019), group task satisfaction (Bruner and Spink, 2011), perceived athletic competency, and perceived global self-worth (Douthitt, 1994). Unfortunately, except that perceived athletic competency and perceived global self-worth were found to be significantly negatively correlated with girls’ exercise adherence, there was no significant correlation between the above individual well-being factors and exercise adherence for children and adolescents. In terms of social well-being factors, including family support for exercise (Faulkner and Michaliszyn, 2021), parental perceived capability to support children’s physical activity and changes in parental support were involved in the study of the relationship between well-being and exercise adherence for children and adolescents (Guerrero et al., 2020). However, only parental perceived capability to support children’s physical activity was shown to be a social well-being factor positively associated with exercise adherence (Guerrero et al., 2020).

For the qualitative studies, one factor of individual well-being factor, self-esteem was involved. Self-esteem was found to be positively associated with exercise adherence (Sunesson et al., 2021), while in terms of social well-being factors, three factors were explored in the study of the exercise adherence, including parental supervision, family support (Birt et al., 2014) and a supportive environment (Sunesson et al., 2021). Of these, a supportive environment and parental supervision were positively associated with exercise adherence. By contrast, family support was negatively associated with exercise adherence in children and adolescents (Birt et al., 2014).

## Discussion

This mini-systematic review highlights the available information on well-being factors that may influence exercise adherence in children and adolescents and how these well-being factors may contribute to exercise adherence in children and adolescents. After systematically filtering and categorizing the extant studies adopting an either quantitative or qualitative approach, only seven studies met the inclusion criteria. The perceived ability of parents to support their children’s exercise behavior (Guerrero et al., 2020), a supportive environment, parental supervision, and self-esteem (Sunesson et al., 2021) were found to be positive factors in promoting exercise adherence in children and adolescents.

Evidence from interventions aimed at enhancing self-esteem might also help reduce depression (Sowislo and Orth, 2013), which coincides with Sunesson’s view that self-esteem and depression are linked in adolescents (Sunesson et al., 2021). When adolescents with depression have high self-esteem, they are more likely to stick to exercise, but when they experience low self-esteem, they will try to avoid potentially harmful experiences. As a consequence, they would not participate in exercise to protect themselves from further harm. A supportive environment is mainly built from encouragement, help from family, friends, personal trainers, or other coaches and the encouragement and support can help adolescents with depression to overcome psychological barriers such as low self-esteem and lack of motivation to exercise and then increase motivation to keep exercising (Searle et al., 2011; Carter et al., 2016; Firth et al., 2016; Jonsson et al., 2017). These findings extended past research focusing on the relationship between social support and adherence to exercise behaviors in adults (Duncan and McAuley, 1993). Although Sunesson et al. (2021) has identified beneficial effects of social support on health and well-being, a relatively large number of research studies are still needed to determine the processes by which social support influences various health promoting behaviors. It is also worth noting that some gray literature that can add some value to the review is not included in the present review. Therefore, future studies might consider adding some gray literature when exploring the association between well-being and exercise adherence.

In contrast with some studies revealing that positive well-being factors was positively related to exercise adherence (Birt et al., 2014; Sunesson et al., 2021), some evidence showed there was no relationship between well-being factors and exercise adherence, and these well-being factors included vigor, self-esteem, predilection, enjoyment, exercise self-efficacy (Alberga et al., 2019), group task satisfaction (Bruner and Spink, 2011), perceived athletic competency, and perceived global self-worth for males (Douthitt, 1994). Some studies even revealed a negative correlation between perceived global self-worth and exercise adherence for females (Douthitt, 1994). These findings underscored the importance of exploring the RQ2 in the present study and uncovered possible moderators that can modulate the influence of well-being on exercise adherence for children and adolescents. Some reasons can explain the inconsistency between results and the presumed positive effect of well-being on exercise adherence and based on these possible explanations, some future directions on moderators can be elucidated. First, even some studies recruited more than 100 children or adolescents, some intervention studies only analyzed data from less than 20 children or adolescents (Faulkner and Michaliszyn, 2021). A limited number of participants could hinder the quality of correlation analysis. Also, extant studies were mainly conducted in western countries, so it remained unclear how well-being factors were related to exercise adherence in Asian countries, such as China. Secondly, measures of exercise adherence varied across the seven included studies, with most of them using self-report measurement and attendance forms for recording. Self-report of exercise adherence might be influenced by social expectancy and false memory of the participants. It is clear that these data do not objectively represent the adherence of the testers. Third, gender could influence the connection between well-being and exercise adherence. For example, Douthitt (1994) found no relationship between well-being and exercise adherence for male students. However, the differences between boys and girls were not explored in the included literature (Birt et al., 2014; Guerrero et al., 2020; Sunesson et al., 2021). One exceptional study that was conducted by Alberga et al. (2019) revealed that potential gender differences between male and female obese adolescents’ predictors of adherence warrant validation in larger studies. Thus, future research should pay more attention to the differentiation of well-being and exercise adherence between children and adolescents of different genders. Fourth, healthy conditions varied for participants from different studies. For example, Sunesson et al. (2021) found self-esteem and a supportive environment were both facilitators for exercise adherence for adolescents with major depressive disorder. It is possible that children or adolescents with mental disorders can benefit from well-being and are facilitated by support and positive effects. However, some adolescents with obesity could find it hard to adopt well-being to increase exercise adherence (Alberga et al., 2019; Faulkner and Michaliszyn, 2021).

For children and adolescents, parental support and supervision could also probably enhance exercise adherence (Birt et al., 2014; Guerrero et al., 2020). However, Faulkner and Michaliszyn, 2021 did not find a positive relationship between family support and exercise adherence. Birt et al. (2014) also found in some families, supporting children to do exercise could arise tension between children and parents. Therefore, how to support and supervise children to adhere to exercise is an essential question to explore in future studies. The degree and ways of support and supervision and traits of children and adolescents should be attended to when providing parental guidance for their children. For example, it is not advisable to exert extreme and much guidance for children with high esteem.

To summarize, the current review systematically explored the relationship between well-being and exercise adherence among children and adolescents. Based on analyzing and reflecting on the included studies, well-being factors that were presumed to impact on exercise adherence were identified (RQ1) and also how exercise adherence was influenced by well-being factors was also investigated (RQ2 and RQ3). The results showed that the positive relationship was not as stable and reliable as presumed. The number of participants, sensitivity and reliability of exercise adherence measurement, gender, and health condition for the children and adolescents might influence the relation between well-being and exercise adherence. Parental support and supervision should also be conducted with caution and adjusted to the differential needs of various children and adolescents.

## Author Contributions

CW and JC developed the conceptual design of the review. JC searched the literature, retrieved the data from the articles, and drafted the whole manuscript. CW checked the data independently and revised the manuscript dramatically. Both authors approved the final version of the manuscript.

## Conflict of Interest

The authors declare that the research was conducted in the absence of any commercial or financial relationships that could be construed as a potential conflict of interest.

## Publisher’s Note

All claims expressed in this article are solely those of the authors and do not necessarily represent those of their affiliated organizations, or those of the publisher, the editors and the reviewers. Any product that may be evaluated in this article, or claim that may be made by its manufacturer, is not guaranteed or endorsed by the publisher.
