# Supplementary material for: On the Relationship Between Well-Being and Exercise Adherence for Children and Adolescents: A Systematic Mini Review
Source: Front Psychol. 2022 May 23;13:900287. doi: 10.3389/fpsyg.2022.900287 (PMC9168894; doi:10.3389/fpsyg.2022.900287)
Supplement: Supplementary file 1 [file Table_1.DOCX]

Appendix A: Quality assessment questions and summary tables.

Questions for quantitative studies

Q1 Sampling method: Was it representative of the population intended in the study?

A. Non-probability sampling (including: purposive, quota, convenience, and snowball sampling) 0

B. Probability sampling (including: simple random, systematic, stratified, cluster, two-stage and multi-stage sampling) 1

Q2 Was a response rate mentioned within the study? (Respond no if response rate was below 60 %)

A. No 0

B. Yes 1

Q3 Was the measurement tool of well-being valid and reliable?

A. No 0

B. Yes 1

Q4 Was the data source primary or secondary?

A. Primary data source 1

B. Secondary data source (survey, not designed for the purpose) 0

Q5 Was exercise adherence examined in the study in a valid and reliable way?

A. No 0

B. Yes 1

Q6 Was the relationship or association between well-being and exercise adherence explored?

A. No 0

B. Yes 1

Table 1: Quality assessment for quantitative studies

| Author(s) and year | Q1 | Q2 | Q3 | Q4 | Q5 | Q6 | Total | Quality |
| --- | --- | --- | --- | --- | --- | --- | --- | --- |
| Alberga et al.,  2019 | 1 | 1 | 1 | 1 | 1 | 1 | 6 | 100% |
| Guerrero et al.,  2020 | 1 | 1 | 0 | 1 | 1 | 1 | 5 | 83.3% |
| Bruner et al.，  2011 | 1 | 1 | 0 | 1 | 1 | 0 | 4 | 66.7% |
| Faulkner et al.,  2020 | 1 | 1 | 1 | 1 | 1 | 1 | 6 | 100% |
| Douthitt et al.,  1994 | 1 | 0 | 1 | 1 | 1 | 1 | 5 | 83.3% |

JBI Critical Appraisal Checklist for Qualitative Research

Reviewer Jiping Chen Date 2022/4/8

Author Sunesson et al Year 2021 Record Number 6

|  | Yes | No | Unclear | Not applicable |
| --- | --- | --- | --- | --- |
| 1. Is there congruity between the stated philosophical perspective and the research methodology? | ☑ | □ | □ | □ |
| 1. Is there congruity between the research methodology and the research question or objectives? | ☑ | □ | □ | □ |
| 1. Is there congruity between the research methodology and the methods used to collect data? | ☑ | □ | □ | □ |
| 1. Is there congruity between the research methodology and the representation and analysis of data? | ☑ | □ | □ | □ |
| 1. Is there congruity between the research methodology and the interpretation of results? | ☑ | □ | □ | □ |
| 1. Is there a statement locating the researcher culturally or theoretically? | ☑ | □ | □ | □ |
| 1. Is the influence of the researcher on the research, and vice- versa, addressed? | ☑ | □ | □ | □ |
| 1. Are participants, and their voices, adequately represented? | ☑ | □ | □ | □ |
| 1. Is the research ethical according to current criteria or, for recent studies, and is there evidence of ethical approval by an appropriate body? | ☑ | □ | □ | □ |
| 1. Do the conclusions drawn in the research report flow from the analysis, or interpretation, of the data? | ☑ | □ | □ | □ |

Overall appraisal: Include ☑ Exclude □ Seek further info □

Comments (Including reason for exclusion)

This research meets my requirements.

Reviewer Jiping Chen Date 2022/4/8

Author Birt et al Year 2014 Record Number 7

|  | Yes | No | Unclear | Not applicable |
| --- | --- | --- | --- | --- |
| 1. Is there congruity between the stated philosophical perspective and the research methodology? | □ | ☑ | □ | □ |
| 1. Is there congruity between the research methodology and the research question or objectives? | ☑ | □ | □ | □ |
| 1. Is there congruity between the research methodology and the methods used to collect data? | ☑ | □ | □ | □ |
| 1. Is there congruity between the research methodology and the representation and analysis of data? | ☑ | □ | □ | □ |
| 1. Is there congruity between the research methodology and the interpretation of results? | ☑ | □ | □ | □ |
| 1. Is there a statement locating the researcher culturally or theoretically? | ☑ | □ | □ | □ |
| 1. Is the influence of the researcher on the research, and vice- versa, addressed? | ☑ | □ | □ | □ |
| 1. Are participants, and their voices, adequately represented? | ☑ | □ | □ | □ |
| 1. Is the research ethical according to current criteria or, for recent studies, and is there evidence of ethical approval by an appropriate body? | ☑ | □ | □ | □ |
| 1. Do the conclusions drawn in the research report flow from the analysis, or interpretation, of the data? | ☑ | □ | □ | □ |

Overall appraisal: Include Exclude □ Seek further info □

Comments (Including reason for exclusion)

This research meets my requirements.
